# Exploring the Crosstalk between Hydrostatic Pressure and Adipokines: An In Vitro Study on Human Osteoarthritic Chondrocytes

**DOI:** 10.3390/ijms22052745

**Published:** 2021-03-09

**Authors:** Sara Cheleschi, Sara Tenti, Marcella Barbarino, Stefano Giannotti, Francesca Bellisai, Elena Frati, Antonella Fioravanti

**Affiliations:** 1 Rheumatology Unit, Department of Medicine, Surgery and Neuroscience, Azienda Ospedaliera Universitaria Senese, Policlinico Le Scotte, 53100 Siena, Italy; sara_tenti@hotmail.it (S.T.); f.bellisai@ao-siena.toscana.it (F.B.); fratielena@unisi.it (E.F.); fioravanti7@virgilio.it (A.F.); 2 Department of Medical Biotechnologies, University of Siena, 53100 Siena, Italy; marcella.barbarino@unisi.it; 3 Center for Biotechnology, Sbarro Institute for Cancer Research and Molecular Medicine, College of Science and Technology, Temple University, Philadelphia, PA 19122, USA; 4 Department of Medicine, Surgery and Neurosciences, Section of Orthopedics and Traumatology, University of Siena, Policlinico Le Scotte, 53100 Siena, Italy; stefano.giannotti@unisi.it

**Keywords:** hydrostatic pressure, adipokines, visfatin, Wnt/β-catenin, mechanical loading, osteoarthritis, obesity, chondrocytes, microRNA, oxidative stress

## Abstract

Obesity is a risk factor for osteoarthritis (OA) development and progression due to an altered biomechanical stress on cartilage and an increased release of inflammatory adipokines from adipose tissue. Evidence suggests an interplay between loading and adipokines in chondrocytes metabolism modulation. We investigated the role of loading, as hydrostatic pressure (HP), in regulating visfatin-induced effects in human OA chondrocytes. Chondrocytes were stimulated with visfatin (24 h) and exposed to high continuous HP (24 MPa, 3 h) in the presence of visfatin inhibitor (FK866, 4 h pre-incubation). Apoptosis and oxidative stress were detected by cytometry, B-cell lymphoma *(BCL)2*, metalloproteinases (*MMPs*), type II collagen (*Col2a1*), antioxidant enzymes, miRNA, *cyclin D1* expressions by real-time PCR, and β-catenin protein by western blot. HP exposure or visfatin stimulus significantly induced apoptosis, superoxide anion production, and *MMP-3*, *-13*, antioxidant enzymes, and miRNA gene expression, while reducing *Col2a1* and *BCL2* mRNA. Both stimuli significantly reduced β-catenin protein and increased *cyclin D1* gene expression. HP exposure exacerbated visfatin-induced effects, which were counteracted by FK866 pre-treatment. Our data underline the complex interplay between loading and visfatin in controlling chondrocytes’ metabolism, contributing to explaining the role of obesity in OA etiopathogenesis, and confirming the importance of controlling body weight for disease treatment.

## 1. Introduction

Obesity represents one of the most influential risk factors for osteoarthritis (OA) incidence, progression, and disability [1]. Its effect on the joint has been traditionally attributed to altered mechanical loading on the articular cartilage of weight-bearing joints [2,3,4,5]; indeed, different mechanical forces in the form of compression, shear stress, and hydrostatic pressure (HP) can affect cartilage homeostasis, leading to irreversible and deleterious effects [5].

Several in vitro studies demonstrated that the application of injurious static HP induced chondrocyte catabolic processes, including degradation of extracellular matrix (ECM) components, production of inflammatory cytokines, oxidative stress, and dysregulation of miRNA expression [6,7,8,9,10,11,12,13,14].

Obesity also increases the risk in developing OA in non-weight-bearing joints, ascribing a prominent role of metabolic factors in the OA pathogenesis [2,15,16]. Interestingly, obesity induces a low-grade chronic inflammatory state mainly through the production of inflammatory mediators, such as adipokines, cytokines, chemokines, and complement factors by white adipose tissue [17]. Adipokines, including adiponectin, leptin, resistin, chemerin, and visfatin, are metabolically active proteins that emerged as crucial regulators of immune system response and chronic inflammation [18,19]. Their critical role in the pathogenesis of immune-mediated rheumatic diseases and degenerative OA has been amply demonstrated [20,21,22,23]. Among them, visfatin is a functionally multi-faceted and ubiquitously protein with insulin-mimetic properties and pro-inflammatory and immunomodulating functions [23,24,25]. Circulating visfatin levels were found higher in patients with OA than those in healthy controls [20,26]; furthermore, pro-inflammatory, catabolic, and pro-degradative effects of visfatin in OA chondrocytes and synovial fibroblasts were revealed [27,28,29,30].

Interestingly, some in vitro studies demonstrated the effect of shear stress or mechanical overloading on adipokine-induced OA damage, exacerbating the loss of chondrocyte homeostasis and accelerating the formation of OA phenotype [31,32,33]. However, the results are still limited and inconclusive, and further investigations to address the characteristics of the interplay between loading and adipokines in the regulation of chondrocytes metabolism and function are needed.

Therefore, the purpose of the present study was to investigate the in vitro role of 3 h of a high continuous HP (24 MPa) in regulating visfatin-induced effects in human OA chondrocyte cultures. In particular, we evaluated the cell viability, the apoptosis ratio, the transcriptional levels of the anti-apoptotic marker B-cell lymphoma *(BCL)2* and of the main extracellular matrix-degrading enzymes, metalloproteinase *(MMP)-3*, *MMP-13*, and of collagen type II alpha 1 chain (*Col2a1*). The production of mitochondrial superoxide anion and the gene expression of antioxidant enzymes (superoxide dismutase *(SOD)-2*, catalase (*CAT*), glutathione peroxidase *(GPx)4*, of nuclear factor erythroid 2 like 2 (*NRF2*)), and of a pattern of miRNA (*mir-27a*, *miR-34a*, *mir-140*, *miR-146a*, *miR-155*, *miR-181a*, and *miR-let7e*) involved in OA pathogenesis were also assessed.

Furthermore, based on our previous results, we analyzed the regulation of the Wnt/β-catenin signaling pathway following HP exposure. To confirm the role of visfatin effects on underlying mechanisms of chondrocytes, cells were pre-treated for 4 h with the visfatin inhibitor FK866.

## 2. Results

### 2.1. HP Regulates Cellular Apoptosis and Cartilage Turnover

**Figure 1:**
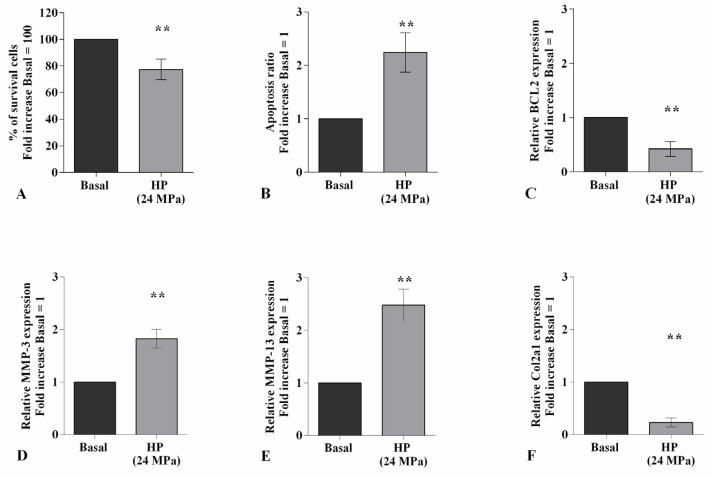
Hydrostatic pressure (HP) exposure regulates chondrocyte metabolism. Human osteoarthritic (OA) chondrocytes were examined at basal condition and after 3 h of high continuous HP (24 MPa). (**A**) Evaluation of cell viability by MTT assay. (**B**) Apoptosis detection performed by flow cytometry analysis and measured with Annexin Alexa fluor 488 assay. Data were expressed as the percentage of positive cells for Annexin-V and propidium iodide (PI) staining. (**C**–**F**) Expression levels of B-cell lymphoma (*BCL2*), metalloproteinase (*MMP)-3*, *-13*, type II collagen (*Col2a1*), analyzed by quantitative real-time PCR. The percentage of survival cells, the ratio of apoptosis, and the gene expression were referenced to the ratio of the value of interest and the value of basal condition, reported equal to 100 or 1. Data were expressed as mean ± standard deviation (SD) of triplicate values. ** *p* < 0.01 versus basal condition.

Figure 1 summarizes the effects of 3 h-application of high continuous HP of 24 MPa on viability, apoptosis ratio, and the regulation of matrix-degrading enzymes, MMP-3, -13, and of Col2a1. The exposure of the cells to HP significantly reduced the percentage of survival and the transcriptional levels of the anti-apoptotic marker *BCL2*, while it raised apoptosis and induced an up-regulation of *MMP-3*, *MMP-13* gene expression, and a decrease of *Col2a1*, in comparison to the basal condition (*p* < 0.01, Figure 1A–F).

### 2.2. HP Influences Oxidative Stress Balance and miRNA Expression Profile

**Figure 2:**
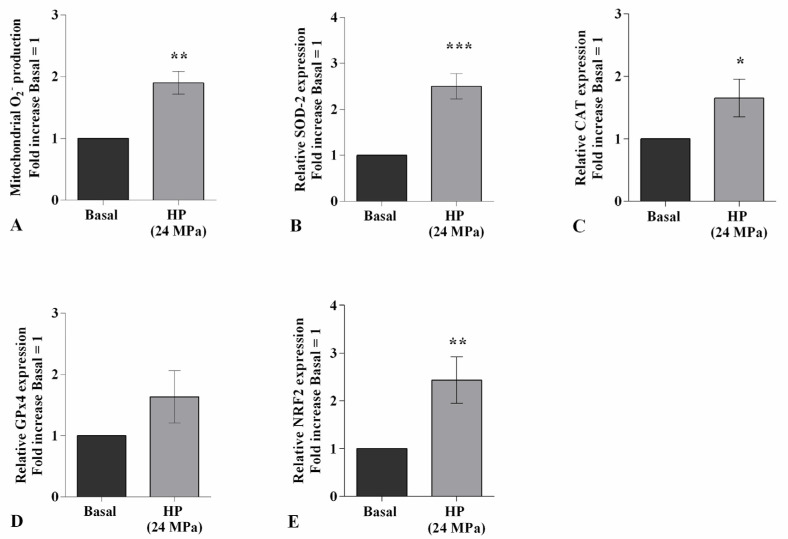
Hydrostatic pressure (HP) exposure regulates oxidative stress balance. Human osteoarthritic (OA) chondrocytes were examined at the basal condition and after 3 h of high continuous HP (24 MPa). (**A**) Mitochondrial superoxide anion production evaluated by MitoSox Red staining at flow cytometry. (**B**–**E**) Expression levels of superoxide dismutase (*SOD*)*-2*, catalase (*CAT*), glutathione peroxidase (*GPx*)*4*, nuclear factor erythroid 2 like 2 (*NRF2*) analyzed by quantitative real-time PCR. The production of superoxide anion and gene expression were referenced to the ratio of the value of interest and the value of basal condition, reported equal to 1. Data were expressed as mean ± standard deviation (SD) of triplicate values. * *p <* 0.05, ** *p <* 0.01, *** *p <* 0.001 versus basal condition.

High HP significantly promoted the production of mitochondrial superoxide anion (*p <* 0.01) and the gene expression of the antioxidant enzymes, *SOD-2* (*p <* 0.001), *CAT* (*p <* 0.05), and of the transcriptional factor *NRF2* (*p <* 0.01), with respect to baseline (Figure 2A–C,E). On the contrary, no detectable changes have been observed in *GPx4* mRNA levels (Figure 2D).

**Figure 3:**
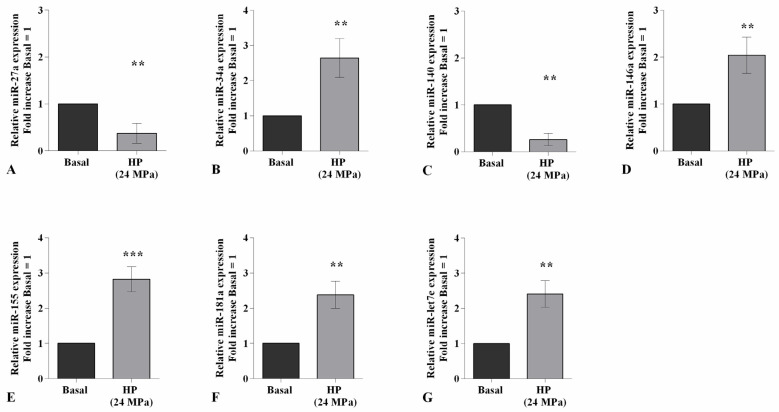
Hydrostatic pressure (HP) exposure modulates miRNA expression. Human osteoarthritic (OA) chondrocytes were examined at the basal condition and after 3 h of high continuous HP (24 MPa). (**A**–**G**) Expression levels of *miR-27a*, *miR-34a*, *miR-140*, *miR-146a*, *miR-155*, *miR-181a*, and *miR-let7e* analyzed by quantitative real-time PCR. The gene expression was referenced to the ratio of the value of interest and the value of basal condition, reported equal to 1. Data were expressed as mean ± standard deviation (SD) of triplicate values. ** *p* < 0.01, *** *p* < 0.001 versus basal condition.

Figure 3 shows the effect of continuous HP of 24 MPa in regulating the gene expression of a pattern of miRNA known to be implicated in OA pathogenesis. The transcriptional levels of *miR-27a* and *miR-140* resulted significantly reduced (*p <* 0.01) in cells exposed to HP in comparison to those at the basal condition (Figure 3A,C). On the other hand, the studied pressurization upregulated, in a significant manner, the gene levels of *miR-34a* (*p <* 0.01), *miR-146a* (*p <* 0.01), *miR-155* (*p <* 0.001), *miR-181a* (*p <* 0.01), and *miR-let7e* (*p <* 0.01) (Figure 3B,D–G).

### 2.3. Visfatin Induces Cellular Apoptosis and Regulates Cartilage Turnover

**Figure 4:**
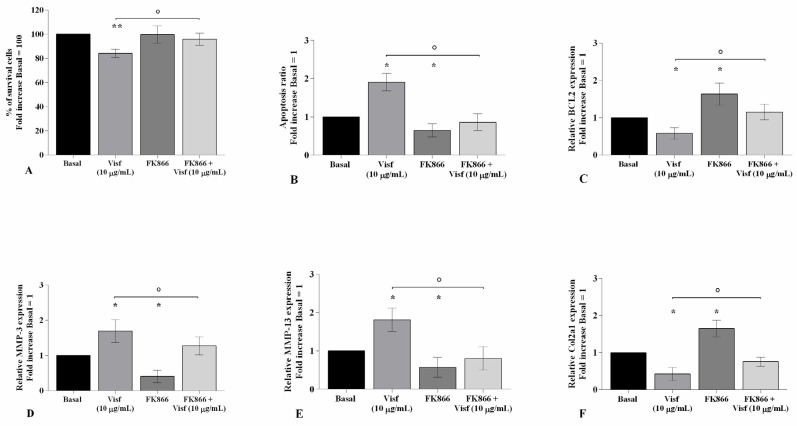
Visfatin regulates chondrocyte metabolism. Human osteoarthritic (OA) chondrocytes were examined at basal condition, after 4 h of pre-incubation with Nicotinamide Phosphoribosyltransferase Inhibitor (FK866, 10 μM), and after 24 h of incubation with visfatin (10 μg/mL). (**A**) Evaluation of cell viability by MTT assay. (**B**) Apoptosis detection performed by flow cytometry analysis and measured with Annexin Alexa fluor 488 assay. Data were expressed as the percentage of positive cells for Annexin-V and propidium iodide (PI) staining. (**C**–**F**) Expression levels of B-cell lymphoma (*BCL2*), metalloproteinase (*MMP*)*-3*, *-13*, type II collagen (*Col2a1*), analyzed by quantitative real-time PCR. The percentage of survival cells, the ratio of apoptosis, and the gene expression were referenced to the ratio of the value of interest and the value of basal condition, reported equal to 100 or 1. Data were expressed as mean ± standard deviation (SD) of triplicate values. * *p <* 0.05, ** *p <* 0.01 versus basal condition. ° *p <* 0.05 versus visfatin.

To confirm the direct effect of visfatin in the modulation of the apoptosis process and cartilage metabolism, OA chondrocytes were incubated for 4 h with 10 μM of visfatin inhibitor (FK866) prior to 24 h of treatment with visfatin (10 μg/mL) (Figure 4). The stimulus of chondrocytes with visfatin significantly reduced the percentage of cell viability (*p <* 0.01) and the expression levels of *BCL2* (*p <* 0.05), while increasing the amount of apoptotic cells (*p <* 0.05), in comparison to baseline (Figure 4A–C). Furthermore, visfatin induced the over-expression of *MMP-3* and *MMP-13* genes (*p <* 0.05) and the downregulation of *Col2a1* (*p <* 0.05) (Figure 4D–F). The incubation of the cells with the FK866 inhibitor significantly counteracted visfatin-induced effects (Figure 4A–F).

### 2.4. Visfatin Modulates Oxidant/Antioxidant System and miRNA Expression Profile

**Figure 5:**
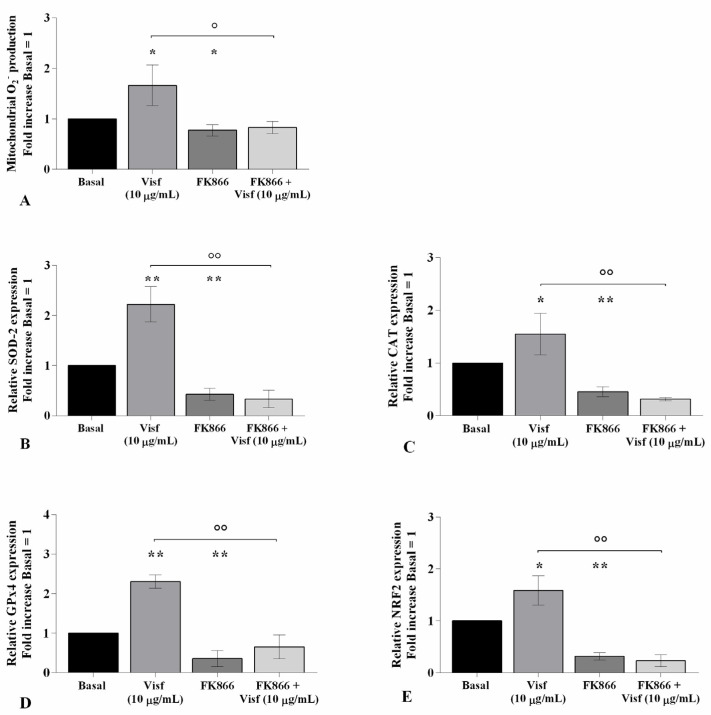
Visfatin regulates oxidative stress balance. Human osteoarthritic (OA) chondrocytes were examined at the basal condition, after 4 h of pre-incubation with nicotinamide phosphoribosyltransferase inhibitor (FK866, 10 μM), and after 24 h of incubation with visfatin (10 μg/mL). (**A**) Mitochondrial superoxide anion production evaluated by MitoSox Red staining at flow cytometry. (**B**–**E**) Expression levels of superoxide dismutase (SOD)-2, catalase (CAT), glutathione peroxidase (GPx)4, and nuclear factor erythroid 2 like 2 (NRF2) analyzed by quantitative real-time PCR. The production of superoxide anion and the gene expression were referenced to the ratio of the value of interest and the value of basal condition, reported equal to 1. Data were expressed as mean ± standard deviation (SD) of triplicate values. * *p <* 0.05, ** *p <* 0.01 versus basal condition. ° *p <* 0.05, °° *p <* 0.01 versus visfatin.

The potential role of visfatin in the regulation of oxidant/antioxidant balance was assessed in visfatin-stimulated chondrocytes pre-treated with a visfatin inhibitor (FK866) (Figure 5).

Flow cytometry and PCR analysis demonstrated a significant increase of mitochondrial superoxide anion production (*p <* 0.05) and of *SOD-2* (*p <* 0.01), *CAT* (*p <* 0.05), *GPx4* (*p <* 0.01), and *NRF2* (*p <* 0.05) transcriptional levels in cells stimulated with visfatin compared to baseline (Figure 5A–E). Conversely, the incubation of the cells with FK866 significantly reduced the ROS production (*p <* 0.05) and antioxidant enzymes’ gene expression (*p <* 0.05, *p <* 0.01) (Figure 5A–E).

Furthermore, pre-treatment of chondrocytes with the inhibitor decreased the ROS release (*p <* 0.05) and the expression of *SOD-2*, *CAT*, *GPx4*, and *NRF2* (*p <* 0.01) induced by visfatin, in comparison to the cells incubated with the adipokine alone (Figure 5A–E).

**Figure 6:**
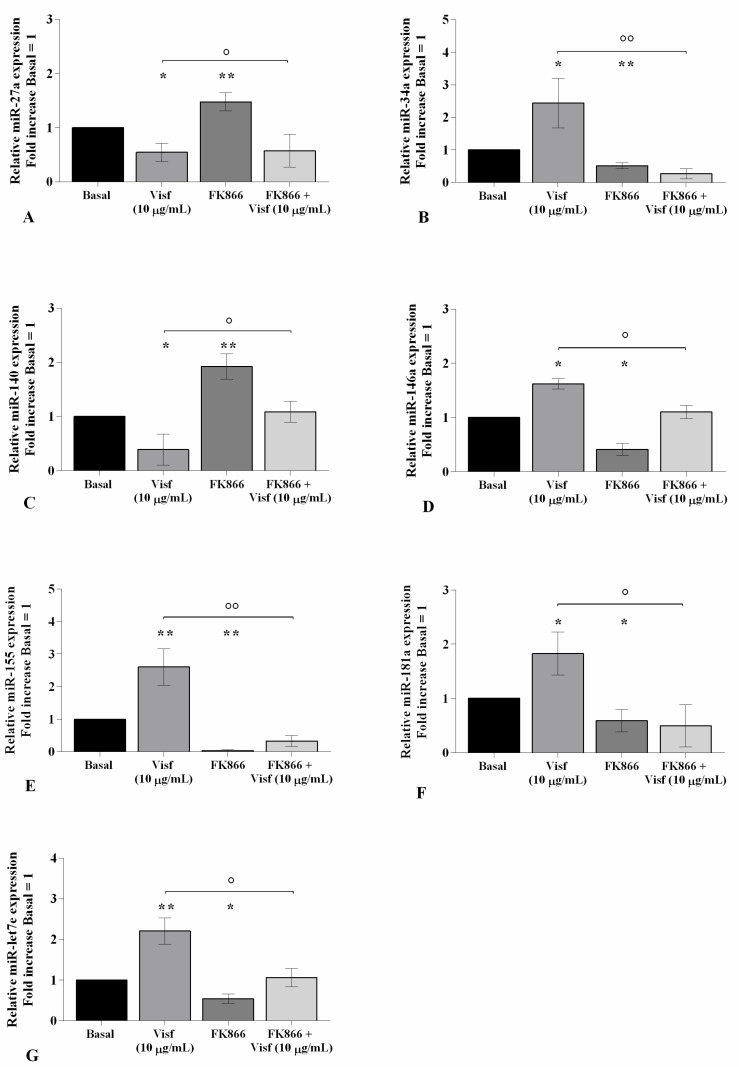
Visfatin modulates miRNA expression. Human osteoarthritic (OA) chondrocytes were examined at the basal condition, after 4 h of pre-incubation with the nicotinamide phosphoribosyltransferase inhibitor (FK866, 10 μM), and after 24 h of incubation with visfatin (10 μg/mL). (**A**–**G**) Expression levels of *miR-27a*, *miR-34a*, *miR-140*, *miR-146a*, *miR-155*, *miR-181a*, and *miR-let7e* analyzed by quantitative real-time PCR. The gene expression was referenced to the ratio of the value of interest and the value of the basal condition, reported equal to 1. Data were expressed as mean ± standard deviation (SD) of triplicate values. * *p <* 0.05, ** *p <* 0.01 versus basal condition. ° *p <* 0.05, °° *p <* 0.01 versus visfatin.

The evaluation of the miRNA expression profile showed a significant down-regulation of *miR-27a* and *miR-140* (*p <* 0.05) gene levels, and an over-expression of *miR-34a* (*p <* 0.05), *miR-146a* (*p <* 0.05), *miR-155* (*p <* 0.01), *miR-181a* (*p <* 0.05), and *miR-let7e* (*p <* 0.01) in visfatin-stimulated cells in comparison to the control cultures (Figure 6A–G). As expected, opposite regulation on the miRNA expression profile was obtained in OA cells incubated with visfatin inhibitor (Figure 6A–G).

### 2.5. HP Increases Cellular Apoptosis and Cartilage Damage Caused by Visfatin

**Figure 7:**
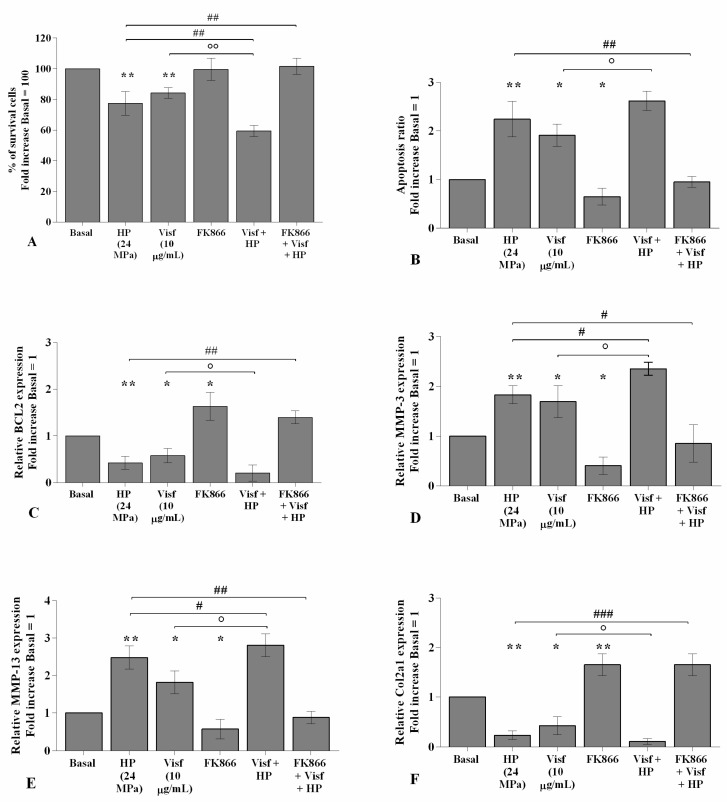
Hydrostatic pressure (HP) exposure exacerbates the effect of visfatin on chondrocyte metabolism. Human osteoarthritic (OA) chondrocytes were examined at the basal condition, after 24 h of incubation with visfatin (10 μg/mL) (4 h of pre-incubation with nicotinamide phosphoribosyltransferase inhibitor (FK866, 10 μM)), and after 3 h of high continuous HP (24 MPa). (**A**) Evaluation of cell viability by MTT assay. (**B**) Apoptosis detection performed by flow cytometry analysis and measured with Annexin Alexa fluor 488 assay. Data were expressed as the percentage of positive cells for Annexin-V and propidium iodide (PI) staining. (**C**–**F**) Expression levels of B-cell lymphoma (*BCL2*), metalloproteinase (*MMP*)*-3*, *-13*, type II collagen (*Col2a1*), analyzed by quantitative real-time PCR. The percentage of survival cells, the ratio of apoptosis, and the gene expression were referenced to the ratio of the value of interest and the value of basal condition, reported equal to 100 or 1. Data were expressed as mean ± standard deviation (SD) of triplicate values. * *p <* 0.05, ** *p <* 0.01 versus basal condition. ° *p <* 0.05, °° *p <* 0.01 versus visfatin. # *p <* 0.05, ## *p <* 0.01, ### *p <* 0.001 versus HP.

Figure 7 shows the implication of HP in regulating visfatin-induced effects on cartilage metabolism; human OA chondrocytes were treated for 24 h with visfatin 10 μg/mL (4 h pre-incubation with 10 μM of visfatin inhibitor, FK866) and, then exposed to 3 h of continuous HP (24 MPa). The concomitant exposure of the cells to visfatin and a cycle of HP significantly exacerbated the regulation on chondrocyte survival, apoptosis, and cartilage turnover caused by the only stimulus with visfatin or HP (Figure 7A–F). In addition, the pre-treatment of chondrocytes with FK866 significantly limited the effects of HP in comparison to what is observed after the pressurization alone (Figure 7A–F).

### 2.6. HP Exacerbates Oxidative Stress Balance Caused by Visfatin

**Figure 8:**
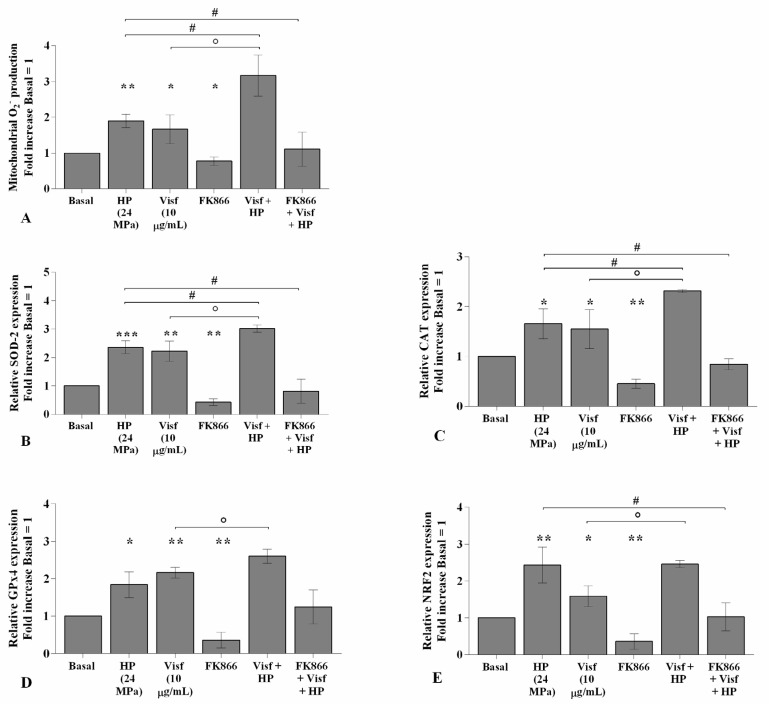
Hydrostatic pressure (HP) exposure increases the effect of visfatin on oxidative stress balance. Human osteoarthritic (OA) chondrocytes were examined at the basal condition, after 24 h of incubation with visfatin (10 μg/mL) (4 h of pre-incubation with nicotinamide phosphoribosyltransferase inhibitor (FK866, 10 μM)), and after 3 h of high continuous HP (24 MPa). (**A**) Mitochondrial superoxide anion production evaluated by MitoSox Red staining at flow cytometry. (**B**–**E**) Expression levels of superoxide dismutase (*SOD*)*-2*, catalase (*CAT*), glutathione peroxidase (*GPx*)*4*, nuclear factor erythroid 2 like 2 (*NRF2*) analyzed by quantitative real-time PCR. The production of superoxide anion and gene expression were referenced to the ratio of the value of interest and the value of basal condition, reported equal to 1. Data were expressed as mean ± standard deviation (SD) of triplicate values. * *p <* 0.05, ** *p <* 0.01, *** *p <* 0.001 versus basal condition. ° *p <* 0.05 versus visfatin. # *p <* 0.05 versus HP.

The effects of visfatin in the regulation of oxidative stress balance were significantly increased (*p <* 0.05) when chondrocytes were also subjected to a high HP of 24 MPa, in comparison to only visfatin stimulus or HP exposure (Figure 8A–E). Furthermore, the activation of oxidant/antioxidant factors was significantly reduced in pressurized cells pre-incubated with FK866 with respect to the only HP exposure (Figure 8A–E).

### 2.7. HP Enhances Visfatin Effect on miRNA Gene Expression Profile

**Figure 9:**
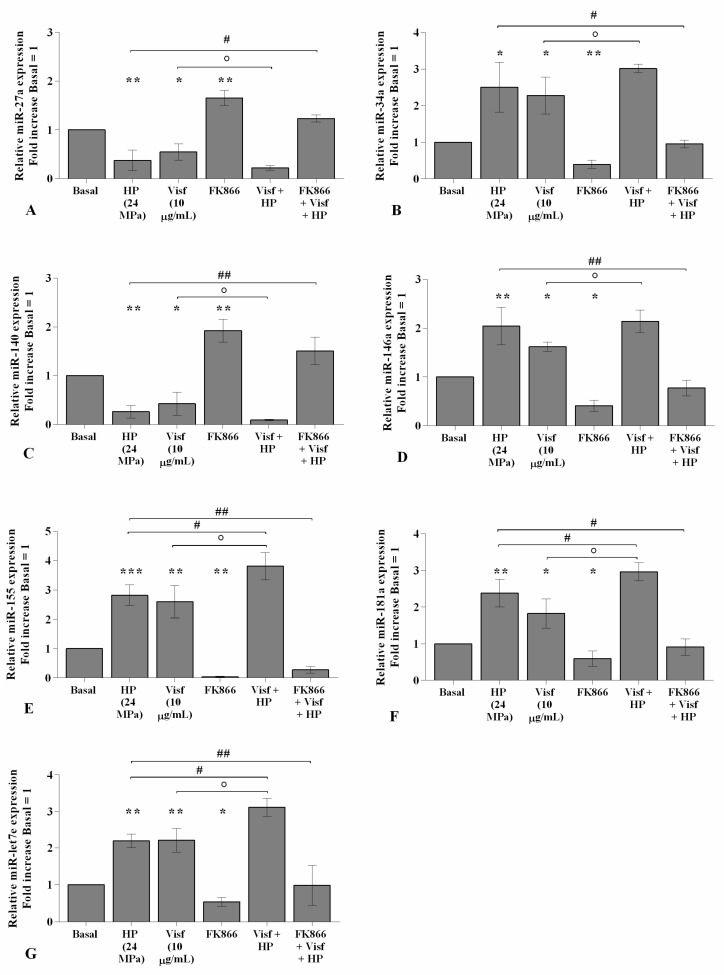
Hydrostatic pressure (HP) exposure increases the effect of visfatin on miRNA expression. Human osteoarthritic (OA) chondrocytes were examined at the basal condition, after 24 h of incubation with visfatin (10 μg/mL) (4 h of pre-incubation with nicotinamide phosphoribosyltransferase inhibitor (FK866, 10 μM)), and after 3 h of high continuous HP (24 MPa). (**A**–**G**) Expression levels of *miR-27a*, *miR-34a*, *miR-140*, *miR-146a*, *miR-155*, *miR-181a*, and *miR-let7e* analyzed by quantitative real-time PCR. The gene expression was referenced to the ratio of the value of interest and the value of the basal condition, reported equal to 1. Data were expressed as mean ± standard deviation (SD) of triplicate values. * *p <* 0.05, ** *p <* 0.01, *** *p <* 0.001 versus basal condition. ° *p <* 0.05 versus visfatin. # *p <* 0.05, ## *p <* 0.01 versus HP.

The effect of visfatin on miRNA expression became significantly more intensive (*p* < 0.05) when OA chondrocytes were also exposed to a cycle of HP with respect to HP or visfatin stimulus alone (Figure 9A–G). In addition, the pre-treatment of the cells with FK866 limited, in a significant manner, the simultaneous effect of visfatin and HP on miRNA regulation in comparison to only visfatin or HP treatments (Figure 9A–G).

### 2.8. HP Influences the Regulation of the Wnt/β-Catenin Pathway Induced by Visfatin

**Figure 10:**
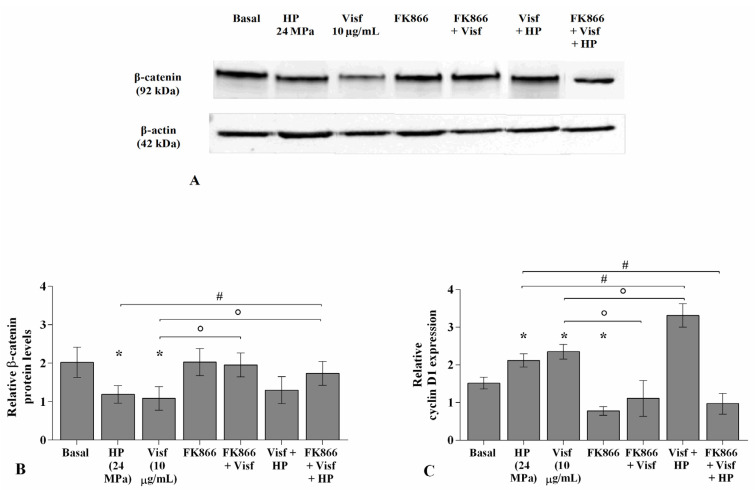
Hydrostatic pressure HP exposure regulates the effect of visfatin on Wnt/β-catenin pathway. Human osteoarthritic (OA) chondrocytes were examined at basal condition, after 4 h of incubation with visfatin (10 μg/mL) (4 h of pre-incubation with nicotinamide phosphoribosyltransferase inhibitor (FK866, 10 μM)), and after 3 h of high continuous (24 MPa) hydrostatic pressure (HP). (**A**,**B**) Representative immunoblotting image and densitometric analysis of β-catenin protein levels by western blot. (**C**) Expression levels of *cyclin D1* analyzed by quantitative real-time PCR. The protein levels and the gene expression were referenced to the ratio of the value of interest and the value of the basal condition, reported equal to 1. Data were expressed as mean ± standard deviation (SD) of triplicate values. * *p <* 0.05 versus basal condition. ° *p <* 0.05 versus visfatin. # *p <* 0.05 versus HP.

Figure 10 describes the regulation of HP on the Wnt/β-catenin pathway activated by visfatin. For the detection of β-catenin protein levels, OA chondrocytes were treated for 4 h with visfatin 10 μg/mL (4 h pre-incubation with 10 μM of visfatin inhibitor, FK866), and then exposed to 3 h of high continuous HP of 24 MPa. Western blot analysis of cell lysates showed the β-catenin band at approximately 92 KDa. The densitometric quantification of the bands revealed that β-catenin protein levels were significantly reduced in OA cells subjected to HP (*p <* 0.05) or following 4 h of visfatin stimulus (*p <* 0.05) in comparison to baseline, while no changes upon the incubation with FK866 inhibitor were observed (Figure 10A,B). A significant increase of β-catenin protein expression (*p <* 0.05) was found in visfatin-treated chondrocytes pre-incubated with FK866 compared to visfatin stimulus alone. Furthermore, FK866 pre-treatment of OA cells simultaneously stimulated with visfatin and HP induced a significant increase of β-catenin expression (*p <* 0.05) with respect to visfatin or HP stimuli alone (Figure 10A,B).

The stimulus of the cells with visfatin or their exposure to HP significantly up-regulated the gene levels of *cyclin D1* (*p <* 0.05) in comparison to those at basal condition, whereas a significant reduction after FK866 incubation was observed (*p <* 0.05) (Figure 10C). FK866 pre-incubation of visfatin-treated chondrocytes significantly reduced the expression of *cyclin D1* (*p <* 0.05) compared to visfatin stimulus alone (Figure 10C). A significant increase of *cyclin D1* expression levels (*p <* 0.05) was observed in OA cells simultaneously exposed to visfatin and HP compared to only visfatin or HP stimulus; the increase of *cyclin D1* was counteracted by the pre-incubation with FK866 inhibitor (*p <* 0.05) (Figure 10C).

## 3. Discussion

Accumulating evidence reported the complex interplay between mechanical loading and adipokines in the development and the progression of OA [3,13,20,34], even if the exact mechanisms underlying this relationship has not completely elucidated and additional studies are required.

The present research aimed to evaluate the potential role of HP in regulating visfatin-induced effects on cartilage turnover, apoptosis, and oxidative stress, and on the modulation of a pattern of miRNA, in human OA chondrocytes. Our experiments were performed using a prototype of the HP system, developed for in vitro cell cultures. In particular, we tested a high continuous HP of 24 MPa, exceeding the range of physiological loading measured in vivo [35]; this pressurization, applied for a period of 3 consecutive hours, approximately reproduced the conditions that occur in the human joints [6,36,37]. Furthermore, to inhibit the enzymatic activity of visfatin, our cultures were pre-treated with FK866, in agreement with other authors [38]. FK866 is a pharmacologic competitive inhibitor that binds the catalytic pocket of nicotinamide and reduces the intracellular NAD content in a time- and concentration-dependent manner [38].

Our results showed the up-regulation of the gene expression of matrix-degrading enzymes, *MMP-3* and *MMP-13*, and the reduction of *Col2a1* in OA chondrocytes exposed to HP or stimulated with visfatin, in agreement with previous studies [6,8,9,13,27,29,39,40,41,42,43].

Furthermore, we first demonstrated that the effect of visfatin on MMPs and Col2a1 was increased when OA chondrocytes were simultaneously subjected to high continuous HP, while the use of visfatin inhibitor limited both visfatin- and HP-induced effects. Evidence from Su et al. [32] partially confirmed our data even if the studies are not comparable. The authors showed a higher increase of cyclooxygenase (*COX*)*-2* gene expression in OA chondrocytes treated for 4 h with resistin and meanwhile exposed to fluid shear stress than the resistin stimulus alone.

The continuous HP of 24 MPa applied to our OA chondrocytes raised the percentage of apoptotic cells, with a concomitant reduction of the gene expression of the anti-apoptotic marker *BCL2*, in agreement with previous studies. Indeed, an increase of apoptosis rate has been reported in human cartilage explants exposed to a single static pressure of 14 MPa for 500 ms under radially unconfined compression [44], and in human or bovine OA chondrocytes subjected to 10 or 20 MPa of loading for a maximum timing of 3 h [13,45,46].

The activation of apoptosis signaling and the reduced expression of the anti-apoptotic marker was also observed when our cell cultures were stimulated for 24 h with visfatin; according to our results, other authors previously revealed the pro-apoptotic effect of this adipokine in endothelial progenitor cells and human OA chondrocytes [13,27,47]. To the best of our knowledge, we found, for the first time, a significant increase of apoptosis exposing OA chondrocytes to visfatin and high continuous HP compared to stimulus with visfatin or HP alone. These results were significantly counteracted upon the pre-incubation with the visfatin inhibitor FK866.

Oxidative stress has been increasingly recognized to be involved in joint damage that occurs in OA. The failure of oxidant/antioxidant balance in chondrocytes determines an altered redox status in favor of catabolic processes, contributing to OA pathogenesis [8,48]. The results of the present study showed an increased production of mitochondrial superoxide anion and an upregulation of the transcriptional levels of the main antioxidant enzymes, *SOD*, *CAT*, *GPx4*, and *NRF2*, after the exposure of OA chondrocytes to HP or visfatin. Similarly, it has been previously reported an excessive ROS production upon the application of 24 h of static compression ranged from 40 to 120 psi or 3 h of static continuous HP of 10 MPa, in porcine and human OA chondrocytes [13,49]. Furthermore, an increase of mitochondrial ROS release and antioxidant enzymes expression was shown in human OA chondrocytes and synoviocytes stimulated with visfatin [29,30]. To our knowledge, this is the first paper showing that the concomitant treatment of OA cultures with visfatin and a cycle of high pressurization significantly exacerbated ROS release and antioxidant enzymes expression; these effects were significantly counterbalanced by FK866 pre-incubation.

An altered expression of some miRNA was associated with the regulation of chondrocyte metabolism, inflammatory response, and oxidative stress during OA damage [29,50,51,52,53]. In this experience, we confirmed the up-regulation of *miR-34a*, *miR-146a*, and *miR-181a* gene expression in OA chondrocytes stimulated with HP, in line with the growing body of evidence [8,13,46,50,53,54,55]. Besides, we first demonstrated the dysregulation of *miR-27a*, *miR-140*, *miR-155*, and *miR-let7e* after the application of this pressurization schedule. Previous studies showed an up-regulation of *miR-27a*, *miR-140*, *miR-146a*, and a decrease of *miR-155* and *miR-181a* gene levels in OA chondrocytes subjected to 3 h of cyclic low HP (1–5 MPa) [8,10].

Moreover, in the present study, the evaluation of visfatin effects on miRNA regulation showed a reduction of *miR-27a* and *miR-140* gene levels, and an increase of *miR-34a*, *miR-146a*, *miR-155*, *miR-181a*, and *miR-let7e* in OA chondrocytes, according to our previous findings in human OA chondrocytes and synoviocytes [27,30].

Finally, we reported a significantly altered expression of the studied miRNA when OA chondrocytes were simultaneously treated with visfatin and exposed to high continuous HP. This combined effect was counteracted by the pre-incubation of the cells with FK866 inhibitor.

The pivotal role of the canonical Wnt/β-catenin signaling pathway in articular cartilage homeostasis and joint disease has been extensively reported [56,57].

Based on our previous findings, in this study we evaluated the regulation of Wnt/β-catenin signaling after a cycle of mechanical loading and/or adipokines stimulus. The exposure of our OA chondrocytes to HP or visfatin stimulus showed a reduction of total β-catenin protein expression. This expression resulted intensified when the cells were simultaneously treated with visfatin and high HP, while it was partially counteracted by the pre-treatment with FK866.

Previous studies found increased protein levels of Wnt-3a and β-catenin in articular cartilage of an injured exercise-induced OA rat model and in OA rat chondrocytes cultures subjected to cyclic mechanical strain with a 0.5 Hz sinusoidal curve at 10% elongation for 8 h/day [58,59]. Recently, Cheleschi et al. showed a reduction of β-catenin protein expression in OA chondrocytes exposed to 3 h of low cyclic sinusoidal HP (1–5 MPa) [8], while its increase was found upon the application of a static continuous HP of 10 MPa [13].

Furthermore, increased protein levels of Wnt-3a and β-catenin were reported after the incubation of human chondrocyte cell lines (C-28/I2 and T/C-28a2) and human OA primary osteoblasts for 24 h with leptin and resistin, respectively [60,61].

Our results seem to be in contrast with the current literature since the apparent non-activation of the signaling pathway following the negative stimuli applied to our cultures. However, this discrepancy could be related to the use, in our experiments, of a non-specific antibody for the assessment of β-catenin expression; indeed, our antibody seemed to be useful in detecting the total β-catenin protein levels, while not able to discriminate between the active non-phosphorylated form and the inactivate phospho-β-catenin labeled for ubiquitination and proteasomal degradation [8,62,63]. In this regard, to confirm the activation of the studied pathway, we also investigated the transcriptional levels of *cyclin D1*, a downstream target gene of Wnt/β-catenin signaling cascade and a central player in cell cycle regulation, cell proliferation, and apoptosis during OA [64]. In this experience, we observed the up-regulation of *cyclin D1* gene expression when OA chondrocytes were exposed to HP and/or to visfatin stimulus. Similar results were found after the exposure of osteoblastic cell lines to 5 h of 3400 microstrains of mechanical loading (2 Hz, 7200 cycles/h) or upon the application of 12 h of 12% cyclical tensile stress at human osteosarcoma cell lines [65,66]. Furthermore, the stimulus of endometrial carcinoma cell lines with visfatin for 24 h induced the expression of *cyclin D1*, which was reduced following FK866 [67].

Finally, we first observed the strong increase of *cyclin D1* gene levels after the combined treatment of chondrocytes with visfatin and HP; the HP effect was reduced by the pre-incubation of the cells with FK866 inhibitor.

## 4. Materials and Methods

### 4.1. Isolation and Culture of Human OA Chondrocytes

Human OA articular cartilage was obtained from femoral heads of five non-obese (body mass index ranging from 20 to 24 kg/m^2^) and non-diabetic patients (two men and three women, age ranging from 63 to 76) with coxarthrosis according to American College of Rheumatology criteria [68], undergoing to hip replacement surgery. OA grades ranged from moderate to severe, and cartilage showed typical OA changes, with the presence of chondrocyte clusters, fibrillation, and loss of metachromasia (Mankin degree 3–7) [69]. The femoral heads were supplied by the Orthopaedic Surgery, University of Siena, Italy. The use of human articular samples was permitted after the authorization of the Ethic Committee of Azienda Ospedaliera Universitaria Senese/Siena University Hospital (decision no. 13931/18), and the informed consent of the donor.

After surgery, cartilage fragments were aseptically dissected from each donor and processed by an enzymatic digestion as previously described [29]. For growth and expansion, cells were cultured in Dulbecco’s modified eagle medium (DMEM) (Euroclone, Milan, Italy) with phenol red and 4 mM L-glutamine (Euroclone, Milan, Italy), supplemented with 10% fetal bovine serum (FBS) (Euroclone, Milan, Italy), 200 U/mL penicillin, and 200 µg/mL streptomycin (P/S) (Sigma–Aldrich, Milan, Italy). The medium was changed every 2–3 days and the cell morphology was examined daily with an inverted microscope (Olympus IMT-2, Tokyo, Japan) [70]. For each single experiment, a cell culture from a unique donor was used.

### 4.2. OA Chondrocytes Exposure to HP

The HP was generated by a unique prototype of pressurization system described in detail by Nerucci et al. [35]; the system has been validated in a number of in vitro studies [6,8,13,71].

In the present study, OA chondrocytes were seeded in Petri dishes (35 × 10 mm^2^) (Euroclone, Milan, Italy) at a starting density of 1 × 10^5^ cells, until they became 85% confluent, in DMEM supplemented with 10% FBS for 24 h. Then, the medium was removed, and substituted with DMEM with 0.5% FBS for the treatment procedure. Petri dishes were completely filled with the culture medium and sealed with a special membrane (Surlyn 1801 Bynel CXA 3048 bilayer membrane, Du Pont, Biesterfeld polychem s.r.l, Milan, Italy), excluding air to avoid implosions due to the presence of air between the membrane and the medium, suitable for preserving a stable environment. The dishes were arranged inside the pressure chamber filled with distilled water at a temperature of 37 °C. Then, the cells were subjected to a high continuous pressure of 24 MPa, for a period of 3 h. Some dishes, used as controls, were maintained in the same culture conditions without receiving any pressurization. Chondrocytes at basal condition and immediately after receiving pressure were collected to perform flow cytometry, quantitative real-time PCR, and western blot analysis.

### 4.3. OA Chondrocytes Treatment

Human OA chondrocytes were plated in 6-well dishes at a starting density of 1 × 10^5^ cells/well until 85% confluence. Human recombinant visfatin (Sigma–Aldrich, Milan, Italy) was dissolved in phosphate buffered saline (PBS) (Euroclone, Milan, Italy), in accordance with the manufacturer’s instructions, and then directly diluted in the culture medium for the treatment in order to obtain the final concentration required.

The cells were cultured in DMEM enriched with 0.5% FBS and 2% P/S, and stimulated for 24 h with visfatin at concentration of 10 μg/mL, according to previous studies [27,29,47]. Some dishes were pre-incubated for 4 h with 10 μM of nicotinamide phosphoribosyltransferase inhibitor, FK866 (Sigma–Aldrich, Milan, Italy) [63].

After the treatment, the cells were recovered and immediately processed to carry out flow cytometry, quantitative real-time PCR, and western blot analysis.

### 4.4. Cell Viability

The viability of the cells was evaluated by MTT (3-[4,4-dimethylthiazol-2-yl]-2,5-diphenyl-tetrazoliumbromide) (Sigma–Aldrich, Milan, Italy) for each experimental condition. The experimental procedure was performed as previously described [30]. The percentage of survival cells was evaluated as (absorbance of considered sample)/(absorbance of control) × 100. Data were reported as OD units per 10^4^ adherent cells.

### 4.5. Apoptosis Detection

Apoptotic cells were measured by using an Annexin V-FITC and propidium iodide (PI) kit (ThermoFisher Scientific, Milan, Italy). OA chondrocytes were seeded in 12-well plates (8 × 10^4^ cells/well) for 24 h in DMEM with 10% FBS, before replacement with 0.5% FBS used for the treatment. The procedure was performed as previously described [13]. A total of 10,000 events (1 × 10^4^ cells per assay) were measured by the instrument. The results were examined with Cell Quest software (Version 4.0, Becton Dickinson, San Jose, CA, USA).

The instrument permitted to discriminate intact cells (annexin-V and PI-negative), early apoptosis (annexin-V-positive and PI-negative), and late apoptosis (annexin-V and PI positives). Cells simultaneously stained with Alexa Fluor 488 annexin-V and PI were considered for the evaluation of apoptosis [72]. The results were expressed as the percentage of positive cells to each dye (total apoptosis).

### 4.6. Mitochondrial Superoxide Anion (·O2-) Assessment

OA chondrocyte were seeded in 12 well-plates (8 × 10^4^ cells/well) for 24 h in DMEM with 10% FCS, before replacement with 0.5% FBS used for the treatment procedure. The procedure has been performed as previously described [13]. A density of 1 × 10^4^ cells per assay (a total of 10,000 events) were measured by flow cytometry and data were analyzed with CellQuest software (Version 4.0, Becton Dickinson, San Jose, CA, USA). Results were collected as the median of fluorescence (AU) and represented the mean of three independent experiments.

### 4.7. RNA Isolation and Quantitative Real-Time PCR

OA chondrocyte were grown and maintained in 6-well dishes at a starting density of 1 × 10^5^ cells/well until they became 85% confluent in DMEM supplemented with 10% FBS, before replacement with 0.5% FBS used for the treatment. After treatment, cells were collected and total RNA, including miRNA, was extracted using TriPure Isolation Reagent (Euroclone, Milan, Italy) according to the manufacturer’s instructions. The concentration, purity, and integrity of RNA were evaluated by measuring the OD at 260 nm and the 260/280 and 260/230 ratios by Nanodrop-1000 (Celbio, Milan, Italy).

Five hundred nanograms of RNA of target genes and miRNA were reverse transcribed by using the QuantiTect Reverse Transcription (Qiagen, Hilden, German) and the cDNA miScript PCR Reverse Transcription (Qiagen, Hilden, German) kits, respectively, according to the manufacturer’s instructions.

Target genes and miRNA were assessed by real-time PCR using QuantiFast SYBR Green PCR (Qiagen, Hilden, German) and miScript SYBR Green (Qiagen, Hilden, German) kits, respectively. Primers used for PCR reactions are listed in Table S1.

All qPCR reactions were achieved in glass capillaries by a LightCycler 1.0 (Roche Molecular Biochemicals, Mannheim, Germany) with LightCycler Software Version 3.5. The reaction procedure for miRNA and target genes has described in detail by our previous studies [13,29].

For the data analysis, the *Ct* values of each sample and the efficiency of the primer set were calculated through LinReg Software [73] and then converted into relative quantities and normalized according to Pfaffl model [74]. The normalization was performed considering actin beta (*ACTB*) for target genes and small nucleolar RNA, C/D Box 25 (*SNORD-25*) for miRNA, as the housekeeping genes [75].

### 4.8. Western Blot

OA chondrocytes at first passage were seeded in Petri dishes (35 × 10 mm^2^) at a starting density of 1 × 10^5^ cells/chamber in DMEM supplemented with 10% FBS for 24 h. After this period, the medium was removed and the cells were cultured in DMEM with 0.5% FBS for the experiment. After treatment, cells were collected and total lysates were obtained with M-PER™ Mammalian Protein Extraction Reagent (Thermo Fisher Scientific, Rockford, IL, USA) containing a protease inhibitor cocktail (Sigma–Aldrich, Milan, Italy). For each experimental condition, ten micrograms were loaded into 10% sodium dodecyl sulphate-polyacrylamide electrophoresis gels and separated by molecular size. Proteins were then transferred to a nitrocellulose membrane and, after blocking step, incubated at 4 °C overnight with mouse monoclonal anti-total β-catenin primary antibody (sc-59737, Santa Cruz Biotechnology, Milan, Italy) (dilution 1:250), and then with secondary goat anti-mouse IgG (H + L)-HRP conjugate antibody (1:5000) (Bio-Rad Laboratories S.r.l., Milan, Italy). The reaction was assessed by chemiluminescence (Bio-Rad Laboratories S.r.l., Milan, Italy). The blots were re-probed with HRP-conjugated β-actin (Sigma-Aldrich, Milan, Italy) used as the loading control. Images of the bands were digitized and the densitometric quantification was performed by Image-J software (LOCI, University of Wisconsin-Madison, Madison, WI, USA). Results were normalized with the relative loading control.

### 4.9. Statistical Analysis

Three independent experiments were carried out and the results were expressed as the mean ± standard deviation (SD) of triplicate values for each experiment. Data normal distribution was evaluated by Shapiro–Wilk, D’Agostino and Pearson, and Kolmogorov–Smirnov tests. Flow cytometry and western blot results were analyzed by ANOVA with a Bonferroni post-hoc test. Quantitative real-time PCR data were evaluated by one-way ANOVA with a Tukey’s post-hoc test using 2^−ΔΔCT^ values for each sample. All analyses were performed through the SAS System (SAS Institute Inc., Cary, NC, USA) and GraphPad Prism 6.1. A *p*-value < 0.05 was defined as statistically significant.

## 5. Conclusions

The results of the present study contribute to increasing knowledge about the complex interplay between HP and visfatin in regulating metabolism in human OA chondrocyte cultures, via the Wnt/β-catenin signaling pathway.

We showed that a cycle of high continuous HP of 24 MPa (3 h), exceeding the physiological loading range measured in in vivo joint, caused cartilage degradation, activated apoptosis signaling, increased oxidative stress, and regulated the expression profile of a miRNA pattern and β-catenin expression and cyclin D1 proteins. Similar and detrimental effects were obtained after 24 h of treatment with visfatin.

Further, the simultaneous exposure of OA cells to visfatin stimulus and high continuous HP seemed to be more effective overall than each single treatment.

Finally, the pre-incubation of the cells with a specific visfatin inhibitor, FK866, reversed both visfatin and HP-induced effects on the analyzed cellular processes.

Taken together, our data support the dual role of obesity in the OA pathogenesis, ascribing a prominent function both to mechanical overloading and the adipose tissue-induced low-grade of chronic inflammation, confirming the importance of controlling body weight in treating the disease.

However, this study reported preliminary results and additional experiments are required to confirm our hypothesis. The implementation of the same analysis on healthy primary chondrocytes could be useful to better understand the involvement of HP and visfatin on chondrocyte homeostasis and, in particular, their relevance in the OA pathogenesis. Furthermore, a deeper analysis of the upstream molecular mechanism responsible for the visfatin-induced effects may contribute to finding out the exact role of mechanical loading in this process. Finally, the use of a specific Wnt/β-catenin inhibitor points out the involvement of the pathway in this complex mechanism.

## Supplementary Materials

Supplementary materials are available online at https://www.mdpi.com/1422-0067/22/5/2745/s1.
