# Supplementary material for: Exploring the Crosstalk between Hydrostatic Pressure and Adipokines: An In Vitro Study on Human Osteoarthritic Chondrocytes
Source: Int J Mol Sci. 2021 Mar 9;22(5):2745. doi: 10.3390/ijms22052745 (PMC7963177; doi:10.3390/ijms22052745)
Supplement: Supplementary file 1 [file ijms-22-02745-s001.pdf]

Table S1. Primers used for quantitative real time PCR.	
**Target Genes**	**Cat. No. (Qiagen)**
BCL2	QT00000721
MMP-3	QT00060025
MMP-13	QT00001764
Col2a1	QT00049518
SOD-2	QT01008693
CAT	QT00079674
GPx4	QT00067165
NRF2	QT00027384
ACTB	QT00095431
CCND1	NM_053056
**miRNA**	**Cat. No. (Qiagen)**
miR-27a	MS00003241
miR-34a	MS00003318
miR-140	MS00003500
miR-146a	MS00003535
miR-155	MS00031486
miR-181a	MS00006692
miR-let7e	MS00031227
SNORD-25	MS00014007

Abbreviations: *MMP-3* = metalloproteinase 1; *MMP-13* = metalloproteinase 13; *Col2a1* = type II collagen; *BCL2* = B-cell lymphoma 2; *SOD-2* = superoxide dismutase 2; *CAT* = catalase; *GPx4* = glutathione peroxidase 4; *NRF2* = nuclear factor erythroid 2 like 2; *ACTB* = Actin Beta; *CCND1* = cicli D1; *miRNA* = microRNA; *SNORD-25* = Small Nucleolar RNA, C/D Box 25.
